# Multiple Sclerosis in Children

**Published:** 2013

**Authors:** Soroor INALOO, Saideh HAGHBIN

**Affiliations:** 1 Associate Professor of Pediatric Neurology, Neonatal Research Center, Pediatrics Department, Shiraz University of Medical Sciences, Shiraz, Iran; 2 Fellow of Pediatric ICU, Assistant Professor of Pediatrics, Neonatal Research Center, Pediatrics Department, Shiraz University of Medical Sciences, Shiraz, Iran

**Keywords:** Multiple Sclerosis, Children, Etiology, Treatment

## Abstract

Multiple sclerosis (MS) is the most important immune-mediated demyelinated disease of human which is typically the disease of young adults. A total of 4% to 5% of MS population are pediatric. Pediatric MS is defined as the appearance of MS before the age of sixteen. About 80% of the pediatric cases and nearly all adolescent onset patients present with attacks typical to adult MS. Approximately 97% to 99% of the affected children have relapsing-remitting MS, while 85% to 95% of the adults experience such condition. MS in children is associated with more frequent and severe relapses. Treatment is the same as adults. We aimed to review the epidemiology, etiology, clinical manifestations, and treatment of MS in children.

## Introduction

**History**

Multiple sclerosis (MS) is the most important immune-mediated demyelinated disease of the human-beings (1). The earliest record of MS dates back to 1837, when Carswell and Cruveilhier separately described the histological lesions of MS in the central nervous system (CNS) (1,2). Frerichs was the first who made the clinical diagnosis of MS in the patients in 1840. A few years later, Kobat identified abnormal oligocloncal bands in the spinal fluids of the MS patients (3). In 1868, Charcot was the first who described associations between the symptoms of MS and the pathological changes in postmortem samples (4). In 1965, National MS Society set up a panel of professionals in order to provide a standard guideline for MS diagnosis (1). In 1916, Dawson provided the definite histological account of the disease (5). Several retrospective studies were published in 1968 and 1990 revealing that some adults with MS had neurological symptoms since adolescence (6). MS is typically the disease of young adults. Pediatric MS is defined as the appearance of MS before the age of sixteen. Its prevalence varies by geographic region ranging from 1.35 to 2.5 per 100.000 in United State to over 248 per 100.000 in western Canada (7,8). About 5% of the patients present before the age of 16 years and less than 1%, before the age of 10 (6,9).

Female to male ratio varies by age and is 0.8:1 under 6 years of age, 1.6:1 between 6 and 10 years, 2.1:1 over 10 years, and 3:1 in adolescence. The most prevalent age of presentation in children is about 12 to13 years (8,10).

**Pathogenesis**

The disease is a dysregulation of immune system that leads to CNS injury. Both genetic susceptibility and environmental factors are required for the initiation of the disease. It is believed that development of MS occurs in the individuals who are genetically susceptible and exposed to triggers during the vulnerability period.

**Genetic Susceptibility**

Monozygote twins studies show a 25-percent concordance for the development of MS. The risk rate in dizygote twins is similar to that of first-degree relatives being almost 2% to 5% (11,12). It has also been shown that certain Human Leukocyte Antigen (HLA) genes including HLA-DRB1, 1501, DQA 0102, and DQB1 0602 are associated with increased risk of MS. HLA DR15 has strongly been associated with early-onset MS (11,13).

**Environmental factors**

Environmental exposure to hundreds of viral and bacterial pathogens such as ebstein barr virus (EBV) is linked to MS. The nuclear antigen of EBV is structurally similar to myelin basic protein, the main component of CNS myelin. T cell activated EBV antigen may damage this protein because of the similarity between these two antigens. Many studies revealed that the adults with MS were almost 100% seropositive for EBV infection, as well (14).

Serologic evidence of past EBV infection was 86% in MS children compared to 64% in normal children (15,16). A 70-percent decreased rate of MS in remote infection with cytomegalovirus (CMV), 70-percent decreased rate in remote infection with herpes virus types 1 in HLA-DRB1 positive patients, and an almost 4-fold decreased rate of the disease in HLA DRB1 negative people are suggestive of gene-environmental interactions (17). Although it is said that vaccination may increase the risk of MS, evidence supports no association between vaccination and MS in adults (18). Also, it has been proved that vaccination against hepatitis B is not associated with increased risk of childhood MS (19).

The disease is more prevalent at more northern latitudes. Migration studies found that individuals who immigrated to high-risk areas in childhood show the risk rate of the new countries rather than the risk of their original countries. Risk of MS among migrants is shown to be influenced by the age at migration with the critical period being prior to the age of 15 years (20).

Some studies have revealed negative correlation between vitamin D (Vit D) levels and the risk of developing MS. Vitamin D is biosynthesized by cutaneous exposure to sunlight. It is known to have immuno-regulatory effects including enhancement of the T-cell activity regulation, up-regulation of anti-inflammatory molecules, and down-regulation of pro-inflammatory cytokines. Some experiments demonstrated the important prenatal effects of vitamin D on the regulation of the normal immune system persisting for the life time (21). Risk of MS is greater in individuals born in May and lower in those born in November. This finding suggests that pregnancy during winter with less sunlight exposure may accompany adverse effect on fetal normal production of vitamin D (22). A small study in pediatric MS found that high circulating levels of vitamin D decreased the attack rates (23). Low levels of vitamin D have been identified as a risk factor of pediatric MS; however, the specific role of this risk factor is still not understood (24). Smoking raises the risk of MS in adults. Similarly, passive smoking has been shown to increase the risk of pediatric MS by 2 times (25). Female obesity at the age of 18 is another risk factor known to increase the risk of MS by 2 folds (26).

**Clinical manifestations**

The disease is characterized by multiple episodes of demyelination in critical areas of CNS (brain, optic nerves, spinal cord) separated with time intervals of at least 30 days.

Clinical features of pediatric and adult MS are similar; however, optic neuritis, isolated brain stem syndrome or symptoms of encephalopathy like headache, vomiting, seizure and/or altered consciousness are more common in children (27).

Approximately 97% to 99% of the affected children and almost 85% to 95% of the adults have relapsing-remitting MS (RRMS). Primary progressive MS (PPMS) is rare in children, and therefore, leukodystrophy, inborn error of metabolism, mitochondrial diseases, and neuromyelitis optica that can be misdiagnosed as MS should be ruled out in every child with continuous disability without specific attacks (28, 29).

**Diagnostic criteria**

Until 2006, Poster criteria were used for the diagnosis of pediatric MS. This was then replaced by “McDonald criteria” being introduced in 2001 and revised in 2005 and 2010 (30-32) (Table1). Core of the criteria is the disseminated lesions based on clinical and finding of the magnetic resonance imaging (MRI) (33). However, nowadays, Poster criteria are only used in countries where MRI is not available (34).

**Table 1:** The 2010 McDonald Criteria for Diagnosis of Pediatric MS (35)*

Clinical presentation diagnosis	Additional data needed for MS
≥ 2 attacks^a^; objective clinical evidence of >2 lesions or objective clinical evidence of 1 lesion with reasonable historical evidence of a prior attack^b^	None^c^
≥ 2atracks^a^; objective clinical evidence of one lesion	Dissemination in space, demonstrated by: ≥ 1 T2 lesion in at least 2 of 4 MS-typical regions of the CNS (periventricular, juxtacortical, infratentorial, or spinal cord)d: or Await a further clinical attack^a^ mplicating a different CNS site
attack^a^: objective clinical evidence of ≥ 2 lesions	Dissemination in time, demonstrated by: Simultaneous presence of asymptomatic gadolinium-enhancing and non enhancing lesions at any time: or A new T2 and/or gadolinium-enhancing lesion(s) on follow-up MRI, irrespective of its timing with reference to a baseline scan; or Await a second clinical attacka
1 attack^a^: objective clinical evidence of 1 lesion (clinically isolated syndrome)	Dissemination in space and time, demonstrated by:For DIS:≥1 T2 lesion in at least 2 of 4 MS-typical regions of the CNS (periventricular, Juxtacortical, infratentorial, or spinal cord)^d^; or Await a second clinical attack a implicating a different CNS site: andFor DIT:Simultaneous presence of asymptomatic gadolinium-enhancing and nonenhancing lesions at any time; or A new T2 and/or gadolinium-enhancing lesion(s) on follow-up MRI irrespective of its timing with reference to a baseline scan; or Await a second clinical attack^a^
Insidious neurological progression suggestive of MS (PPMS)	One year of disease progression (retrospectively or prospectively determined) plus 2 of 3 of the following criteria^d^: 1. Evidence for DIS in the brain based on 1 T2 lesions in the MS characteristic (periventricular, juxtacortical, or infratentorial) region 2. Evidence for DIS in the spinal cord based on 2 T2 lesions in the cord 3. Positive CSF (isoelectric focusing evidence of oligoclonal bands and/ or elevated IgG index)

If the Criteria are fulfilled and there is no better explanation for the clinical presentation, the diagnosis is “MS”; if suspicious, but the Criteria are not completely met, the diagnosis is “possible MS”; if another diagnosis arises during the evaluation that better explains that clinical presentation, then the diagnosis is “not MS.”

*MS= multiple sclerosis; CNS= central nervous system; MRI= magnetic resonance imaging; DIS= dissemination in space; DIT dissemination in time; PPMS= primary progressive multiple sclerosis; CSF= cerebrospinal fluid; IgG= immunoglobulin G.*

a An attack (relapse; exacerbation) is defined as patient reported or objectively observed events typical of an acute inflammatory demyelinating event in the CNS, current or historical, with duration of at least 24 hours, in the absence of fever or infection. It should be documented by contemporaneous neurological examination, but some historical events with symptoms and evolution characteristic for MS, but for which no objective neurological findings are documented, can provide reasonable evidence of a prior demyelinating event. Reports of paroxysmal symptoms (historical or current) should, however, consist of multiple episodes occurring over not less than 24 hours. Before a definite diagnosis of MS can be made, at least 1 attack must be corroborated by findings on neurological examination, visual evoked potential response in patients reporting prior visual disturbance, or MRI consistent with demyelination in the area of the CNS implicated in the historical report of neurological symptoms.

b Clinical diagnosis based on objective clinical findings for 2 attacks is most secure. Reasonable historical evidence for 1 past attack in the absence of documented objective neurological findings, can include historical events with symptoms and evolution characteristics for a prior inflammatory demyelinating event; at least 1 attack, however, must be supported by objective findings.

c No additional tests are required. However, it is desirable that any diagnosis of MS be made with access to imaging based on these criteria. If imaging or other tests (for instance, analysis of CSF) are undertaken and are negative, extreme caution needs to be taken before making a diagnosis of MS, and alternative diagnoses must be considered. There must be no better explanation for the clinical presentation, and objective evidence must be present to support a diagnosis of MS.

d Gadolinium-enhancing lesions are not required; symptomatic lesions are excluded from consideration in subjects with brain stem or spinal cord syndromes.

McDonald criteria should not be used as diagnostic criteria for children presenting with encephalopathy and multifocal neurological deficit (35). 

A total of 15% to 20 % of MS children present with the symptoms similar to acute disseminated encephalomyelitis (ADEM). Most of such children are younger than 11 years.

Typical lesions of ADEM are poorly demarginated in deep and sub-cortical white matter and generally bilateral. Children with early onset MS (at age younger than 11 years) have larger and less well-defined lesions when compared with typical brain lesions in teenagers and adults. Application of McDonald criteria in these children with such lesions, specially with symptoms of encephalopathy, is thus inappropriate and continuous follow-up is needed to confirm both clinical and MRI findings for the diagnosis of MS (36,37). Current consensus criteria for MS diagnosis in children with an ADEM-like first attack need confirmation by two or more non ADEM-like attacks or one non ADEM-like attack followed by clinically silent lesion (38). Also in children presented with insidious neurological progression and considered as primary progressive MS (PPMS), criteria is different (Table 1) (39-41).

Clinically isolated syndrome (CIS) is a single attack compatible with MS and one type of symptoms such as optic neuritis. About 80% of pediatric MS cases present with attacks similar to adult CIS (42-44). Episodes of CIS are diagnostic and therapeutic challenges. Majority of the children do not experience the second attack. In such a situation, clinical investigations including brain MRI, CSF analysis and other laboratory studies can differentiate the high-risk and low-risk groups regarding recurrence. A series of factors including age of 10 years and older, optic nerve lesion, and typical MS lesions in the MRI (typical well-defined lesions in periventricular or subcortical areas) are believed to increase the risk of recurrence (45). Spinal cord lesions and acute mental status change may-on the other hand- decrease the risk of second attacks.

**MRI findings in MS**

1. Multiple well-demarcated lesions in the periventricular, subcortical, infratentorial, and spinal cord white matter that are better diagnosed on T2 weighted sequences and T2 fluid attenuated inversion recovery (FLAIR). FLAIR sequences are the most sensitive in the evaluation of lesions especially in the periventricular lesions.

2. “Black holes” on T1 weighted sequences representing complete tissue loss due to previous inflammatory lesions. 

3. Enhancement of active area of inflammation and blood-brain barrier compromise on T1 gadolinium contrast sequences.

Pediatric MS accompanies more T2 bright lesions in the posterior fossa and more gadolinium enhancement lesions than in adults. Lesions are more reversible on follow-up imaging in children and suggest a better recovery course (41). MS in children also appears to be a highly inflammatory disease with more frequent relapses compared to adults (43).

**CSF analysis**

A CSF analysis is defined to be positive for MS if oligoclonal IgG bands (OCBs) or increased IgG index are present. The 2001 and 2005 McDonald criteria mentioned that positive CSF and new lesions on serial MRI were enough for confirmation of the diagnosis of MS even in patients with a single clinical attack (30). However, this was not considered in the diagnosis by the 2010 criteria except for the diagnosis of PPMS. 

CSF profile in pediatric MS is different from adults. Positive OCBs, elevated IgG index, and pleocytosis have been reported in 8% to 92%, 64% to 75%, and 33% to 73% of the MS children, respectively (46). 

**Evoked potentials**

Visual evoked potentials (VEP) and somatosensory evoked potentials (SSEP) provide supportive evidence for demyelination in the optic nerve, and brain stem or spinal cord and help diagnosis of MS; however, their utility in confirming the diagnosis is not yet established (47,48).

## Treatment

**Acute attack: **Glucocorticoids are the main treatment of acute attacks. Intravenous (IV) pulse of methylprednisolone (20 to 30 mg/kg, daily, for five days) is a typical regimen. No further glucocorticoid is needed if patients recover completely. However, oral prednisolone starting with 1 mg/kg/day and decreasing by 5 mg every two days is used for the patients with residual disability. For patients who experience recurrence during glucocorticoid tapering, repeated treatment with IV methylprednisolone with the same doses is suggested. Intravenous immune globulin (IVIG) (400 mg/kg/day for 5 days) can be used for the patients refractory to glucocorticoids or during febrile illnesses when infection is suspected or if there is contraindication for steroid consumption (49,50). Plasmapheresis (plasma exchange) is reserved to be used in severe fulminant relapses refractory to treatment with steroid or intravenous immunoglobulin (51). 

**Long term treatment: **Immunomodulating agents including interferon beta drugs (interferon beta-1a and 1b) and glatiramer acetate are used to prevent relapses or progression of MS. In adults, these agents reduce the recurrence rate of MS by 30%. However, no randomized controlled trials have been done to elucidate the effect of these treatments on MS children. Nevertheless, different drug regimens in pediatric patients have been shown to decline the recurrence rate and slow the progression of MS.

**Drug dose:**

- Interferon beta-la (Avonex): 30 mcg/ intramuscular (IM)/ once weekly;
- Interferon beta-1a (Rebif): 22 or 44mcg/ subcutaneous (SC)/ three times a week;
- Interferon beta-1b (betaferon): 8 million international units/ SC/ every other day;
- Glatiramer acetate: 20 mg/ SC/ daily.

The recommended doses of these drugs are similar in both adults and adolescents heavier than 50 kg; but, for children less than 10 years of age, the dose is calculated based on the child weight in kilograms divided by 50kg and multiplying the results by adult dose (52-55). 

## Side effects

**1. Glatiramer acetate**

The most common side effect is transient skin reactions at the site of the injection that resolve with continuing the treatment. Chest pain and flushing are also of the other transient complications occurring immediately after the injection.

**2. Interferon**

The most common side effects are flue-like syndrome, headache, transient elevation of the liver enzymes, leucopenia, anemia, thrombocytopenia, and thyroid dysfunction mandating regular monitoring during the treatment courses. Abnormalities often resolve with dose reduction.

Interferon beta-la (Avonex) is often preferred in children because of its weekly injection dose. It is also recommended for the patients with less frequent attacks, low-density brain lesions, and no disability. 

Glatiramer acetate, Betaferon, and Rebif are suggested for the patients with the history of more than one attack in a year, those with fixed disabilities, and those with many brain lesions (plaque) or atrophy on MRI. 

Interferon beta treatment is better to be avoided in the patients with depression because of exacerbating depression symptoms. In such patients, glatiramer acetate is preferable. Besides, it is recommended for the patients who experience liver enzymes abnormalities following interferon injection.

Patients’ clinical signs and symptoms as well as their neuroimaging parameters should be regularly monitored. The international pediatric MS study group suggests baseline neurological examination followed by examinations on the first, third, and sixth months of therapy and every six months, afterwards. Annual MRIs are considered by many neurologists. 

For the patients with progressive symptoms or diffused lesions on brain MRI compared to the baseline scan, the therapeutic regimen should be changed and a more aggressive treatment should be started (55). 

Refractory MS is defined as the occurrence of three or more relapses in a 12-month period, significant increase in MRI lesions, or progression of disability in spite of immunomodulatory therapy.

If the patients do not respond to Avonex therapy and show disease progression, the treatment regimen can be changed to higher doses of interferon beta (Rebif or Betaseron) or glatiramer acetate. Poor responses to high dose interferon can be replaced by glatiramer acetate. However, for patients with progressive diseases on high-dose interferon beta or glatiramer acetate, immunosuppressive medications such as mitoxantrone (anthracycline agent) or cyclophosphamide or rituximab or daclizumab can also be added (56,57). 

Although no evidence supports the effectiveness of these drugs in pediatric MS, in fulminant or aggressive MS mitoxantrone can be used with dose of 12 mg/m2, IV, every 3 months for 2 years. Lifelong cumulative dose should not be more than 140 mg/m2. Considering its cardiac toxicity, use of mitoxantrone should be limited to pediatric patients with rapidly advancing disease and no response to other therapies.

Natalizumab- a recombinant humanized monoclonal antibody- has been recently approved as monotherapy in adults with RRMS but not for children yet. In only one pediatric study carried out in Italy, natalizumab (300 mg/day, every 28 days) was used in 19 children with severe active MS. During a 15-month period of followup relapses and MRI lesions suppressed in all patients and no serious side effects were reported. However, this medication has not yet been approved to be used in pediatric MS patients (58). High-dose cyclophosphamide followed by autologous haematopoietic stem cell transplantation (AHSCT) is also a new treatment option for aggressive and refractory MS and showed progression-free survival proportion between 50 and 77% in short term in adult (59). Oral agents including fingolimod and teriflunomide have been approved by FDA to be used in adult relapsing – remitting MS; however, they have been accompanied by serious side effect including lethal herpetic infection, cancer, and cardiac death. There is no study to evaluate the use of fingolimod in pediatric MS population (51).

## Supportive and symptomatic therapy

Symptomatic treatments help improve the quality of life in the patients. Fatigue is one of the most common complaints of the MS cases which may be due to the demyelinated nerve fibers, depression, or daily stresses at school or work. Depressed patients should be referred to psychiatrists and receive treatment. However, the most common cause of fatigue is the disease itself (60). Occupational therapy may be useful to relieve it. Medications such as amantadine, modafinil, fluoxetin, and methylphenidate are also helpful. Amantadine and modafinil are typically the first-line treatment options. The recommended dose of amantadine is 2.5 mg/kg, twice a day (maximum dose of 150 mg) for children under 40 kg or younger than 10 years of age and 100 mg, twice a day for the older children and adults. The dose can be increased to total 400 mg if needed.Modafinil is started with the dose of 50 to100 mg once a day for children under 10 years and 100 mg/day for older ones. The dose is increased to 300 mg/day if needed (61).

Methylphenidate and other amphetamines are given at doses similar to those used for attention deficit hyperactive disorder (62).

Fluoxetine is started at the dose of 10 mg every other day for 2 weeks and then increased to 20 mg/day for children under 10 years and up to 40 mg/day for adolescents. Fluoxetine should be used cautiously in depressed patients because it sometimes increases the suicide tendency. Child psychiatrists should therefore be consulted in children and adolescents with evidences of mood disorder presentations.

Cognitive impairments are common in adult with MS and may also bee seen in MS children. The severity of such symptoms depends on the duration and severity of MS and may involve language, visuomotor integration, and verbal and visual memory (62).

## Prognosis

Disability due to MS is highly variable in the patient; but, the progression is usually very slow. A total of 97% to 99% of the pediatric MS population experience relapsing, remitting courses and only 1% to 2% of childhood-onset MS has progressive course.

Several studies showed that a younger age at MS onset is associated with slower disease progression. However, patients with pediatric-onset MS developed disability at younger ages compared to those with adult-onset MS because of a longer duration of disease and early-onset presentation of the disease.
